# Encapsulated Butyric Acid and Zinc as a Feed Additive for Lambs Abruptly Transitioned to a Grain-Based Diet †

**DOI:** 10.3390/biology13060457

**Published:** 2024-06-20

**Authors:** Forest L. Francis, Thiago Lauro Maia Ribeiro, Doug LaFleur, Jerilyn E. Hergenreder, Zachary K. Smith

**Affiliations:** 1 Department of Animal Science, South Dakota State University, Brookings, SD 57007, USA; forest.francis@sdstate.edu (F.L.F.); thiagolauro.maiaribeiro@sdstate.edu (T.L.M.R.); 2 Kemin Industries, Inc., Des Moines, IA 50317, USA; doug.lafleur01@gmail.com (D.L.); jerilyn.hergenreder@kemin.com (J.E.H.)

**Keywords:** feedlot, gut health, histology, morphology, rumen, ruminant

## Abstract

**Simple Summary:**

During diet transition periods, ruminal acidosis can have negative effects on gut health and performance of ruminants and investigating feed additives that may help alleviate some of these negative effects is critical. Butyric acid and zinc have been studied as feed additives that can improve gastrointestinal health in ruminants and may be suitable to help decrease negative effects of ruminal acidosis. Thus, a study was conducted to evaluate if a partially encapsulated butyric acid and zinc feed additive was effective at improving growth performance, carcass characteristics, and gastrointestinal health in feedlot lambs abruptly transitioned to a 100% concentrate diet.

**Abstract:**

Butyric acid is attributed to gastrointestinal epithelial development and health and two studies were conducted to determine if supplementing encapsulated butyric acid and zinc (BZ) in lambs abruptly transitioned to a finishing diet has effects on growth performance, efficiency of dietary net energy utilization, rumen morphometrics, small intestinal histology, and carcass traits. Polypay wethers (n = 84; initial shrunk body weight = 38.8 kg ± 4.8 kg) were used in a randomized complete block design study. Wethers were abruptly transitioned from a high roughage-based diet to a 100% concentrate-based diet and dietary treatments were 0 or 2 g BZ/kg of diet dry matter. Study 1 evaluated growth performance and carcass traits of lambs over a 59.5 d feeding period, and Study 2 evaluated changes in rumen morphometrics and small intestine histology in serial harvested lambs. Wethers supplemented with BZ had increased body wall thickness, decreased calculated boneless closely trimmed retail cuts, and decreased red meat yield (*p* ≤ 0.03) compared to non-supplemented wethers. Linear effects (*p* ≤ 0.01) for harvest date were observed for most rumen and small intestine measurements. Supplementing wethers with BZ did not improve growth performance, carcass traits, or rumen and small intestine measurements. The effects of BZ supplementation on fat deposition in ruminants should be further investigated.

## 1. Introduction

Postnatal development of the rumen and intestinal epithelium is stimulated by short chain fatty acids (SCFAs). Of the SCFAs attributed to epithelial development, butyric acid has the greatest effect on rumen and intestinal epithelial cell proliferation [1,2]. In ruminants, the primary butyrate-producing bacteria are represented by *Butyrivibrio fibrisolvens* strains [3]; however, past research has investigated the effects of supplementing exogenous butyrate on gastrointestinal health in ruminants. In dairy calves, supplementing butyrate has shown beneficial results in growth performance and gastrointestinal health including improved daily gain, decreased fecal scouring days, increased ruminal papillae size, elevated mitotic index, and lower apoptotic index [4,5]. The addition of sodium butyrate into suckling lamb diets has been reported to increase dry matter intake, average daily gain, hot carcass weight, dressing percentage, and rumen papillae length compared to control lambs [6].

Zinc is a mineral that is essential for a plethora of metabolic and enzymatic processes, and is essential for DNA, RNA, protein synthesis, and cell division [7]. The suggested minimum zinc requirement for growing lambs is 20 mg/kg of dry matter; however, additional supplementary zinc may be beneficial to support gastrointestinal epithelial health [8]. Supplementary zinc has been reported to improve intestinal permeability and upregulate expression of tight junction proteins in the intestines [9,10]. Finishing lambs supplemented with differing zinc sources showed improvements in final body weight, average daily gain, feed conversion, dry matter digestibility, and jejunal villus width compared to non-supplemented lambs [11]. Additionally, Esfiokhi et al. [11] reported that lambs supplemented with organic zinc sources had improved duodenal villus width and ileal villus height compared to non-supplemented lambs.

In ruminants, if carbohydrate supply is increased abruptly, like during periods of grain engorgement, the total supply of SCFAs and lactate increases which can cause acidosis [12]. Grain-induced acidosis in dairy cows has been reported to reduce the total depth of the rumen epithelium, decrease cellular junctions, and slough the stratum corneum layer of the rumen epithelium in as little as a week following transition to a high grain diet [13]. Additionally, in high-concentrate-fed dairy cows, the abundance of pro-apoptotic proteins is increased while the abundance of anti-apoptotic proteins is decreased, indicating apoptosis of rumen epithelial cells which is associated with increased permeability of the rumen epithelium [14]. Furthermore, during grain-induced acidosis, considerable portions of starch may bypass rumen fermentation and enter the lower digestive tract and trigger barrier dysfunction, lipopolysaccharide translocation, and inflammation [15]. Thus, grain engorgement that may lead to acidotic conditions has the potential to induce both rumen and lower gastrointestinal tract barrier dysfunction in ruminants fed high-concentrate diets.

Both butyric acid and zinc supplementation have been investigated as potential strategies to mitigate the negative effects of ruminal acidosis. Supplemental butyric acid has been reported to induce rumen papillae growth by decreasing apoptosis in the rumen epithelium by down-regulating expression of insulin-like growth factor binding protein 3 [5,16]. In dairy cows subjected to experimental acidosis via a feed restriction protocol, supplementary zinc improved ileal villus height and ileum mucosal surface area compared to non-supplemented controls [17].

Based on previous research, the addition of butyric acid and zinc to ruminant diets has the potential to improve rumen and intestinal health and subsequently improve performance metrics. Thus, these two studies were conducted to determine if a time-released encapsulated butyric acid and zinc (BZ) supplement fed to feedlot lambs abruptly transitioned to a grain-based finishing diet influences growth performance, efficiency of dietary net energy (NE) utilization, rumen morphometrics, small intestine histology, and carcass characteristics. Our hypothesis was that supplemented lambs would have improved growth performance and carcass characteristics resulting from improved rumen and gastrointestinal health.

## 2. Materials and Methods

All procedures involving the use of animals in these experiments were approved by the South Dakota State University Institutional Animal Care and Use Committee (Approval #2101-004E).

### 2.1. Dietary Treatment

For both trials conducted the dietary treatment was identical and used the same group of lambs. Dietary treatments included:

1. 0 g BZ/kg diet dry matter (CON);
2. 2 g BZ/kg diet dry matter (BZ).

The level of BZ included in the supplemented diet was determined from a previous beef feedlot study conducted in our lab which evaluated increasing doses of BZ in finishing diets [18]. The BZ was manufactured with proprietary spray freezing technology, microencapsulating butyric acid and zinc in a lipid matrix that allows for a timely release throughout the entire gastrointestinal tract. The test product was commercially formulated to contain 25% butyric acid and 10% zinc oxide. No monensin sodium was fed over the course of the experiment, and decoquinate (Deccox^®^, Zoetis Services LLC, Parsippany, NJ, USA) was fed at 77.22 mg/kg of complete diet (DM basis) for the prevention of coccidiosis. All diets were fortified with vitamins and minerals to exceed nutrient requirements for growing lambs [8].

Total mixed rations for both CON and BZ treatments were manufactured in two manufacturing events at the SDSU Feed Mill in Brookings, SD, on 30 August 2021 and 14 October 2021. The manufactured rations were delivered to the SDSU Sheep Unit in Brookings, SD, and stored in upright hopper bins.

### 2.2. Sheep Feeding and Management

Polypay wethers (n = 84; initial shrunk body weight [BW] = 38.8 kg ± 4.8 kg) from the SDSU sheep flock were sourced for the trials. Wethers (n = 80) were blocked (n = 8) by initial shrunk BW into treatment pens (n = 16; n = 5 wethers/pen). At allotment, a subset of lambs (n = 4; initial shrunk BW = 39.1 kg ± 0.4 kg) closest to the median weight of all lambs were harvested at the SDSU Meat Laboratory and used as a baseline for empty body, histological, and carcass measures.

All lambs were comingled and received a common high roughage diet (Table 1) for 89 d prior to initiation of test diets at study onset. On 27 August 2021, individual BWs were recorded for allotment purposes. On the morning of 30 August 2021, lambs were individually weighed again, paint branded with the home pen number, and allotted into treatment pens. Upon study initiation, lambs were immediately transitioned to their grain-based treatment diets (Table 2) without a step-up period. Wethers were fed in 18 m^2^ dirt pens with continuous flow stainless steel water troughs that were shared between pens within blocks. Feed was offered in waterproof range feeders placed in each pen, and pans were managed to be slick allowing ad libitum access to feed, with minimal day to day variation in feed deliveries. Initial offering of feed was 2% (as-fed basis) of initial shrunk BW; feed delivered was increased by 0.1 kg/hd/d (as-fed basis) for d 1–4 and 0.2 kg/hd/d (as-fed basis) for d 5–7. For d 1–7, delivered feed was increased from 2% to 4.7% (as-fed basis) of initial shrunk BW to subject the lambs to a dietary transition challenge. During the challenge, lambs were fed once daily at 0700 h and feedings were managed to be slick at 0630 h. Following the challenge, delivered feed was increased by 0.5 to 1.5 kg/hd depending on previous feeding behavior; lambs were fed twice daily at 0700 h and 1900 h and pans were managed to be slick at 0630 h and 1830 h. Feed was delivered to individual pens in buckets and deliveries were weighed to 227 g increments on a digital scale (4.54 g scale break).

On study d 7, 14, and 21, 56, and 63, lambs were weighed, and one lamb was randomly selected from each pen (n = 16/period) for harvest the following day at the SDSU Meat Laboratory for collection of empty body weight, liver abscess severity, rumen and small intestine samples for histological analysis, and carcass data collection.

### 2.3. Study 1—Growth Performance, Efficiency of Dietary NE Utilization, and Carcass Data

#### 2.3.1. Management

Study 1 was an evaluation of growth performance, efficiency of dietary net energy utilization, and carcass traits of lambs subjected to the previously discussed dietary treatments. This study utilized only the 32 lambs that remained following the d 21 weigh and harvest dates. Because of the metabolic effects of the feed-induced acidosis challenge, for the first 21 d of the experiment lamb average daily gain (ADG) and feed conversion efficiency (G:F) for many pens was negative, and it was determined that the overall effectiveness of the encapsulated butyric acid and zinc product should be evaluated over a longer period of time to determine its effectiveness on growth performance outcomes. Thus, the 32 hd of lambs (n = 2 hd/pen; n = 16 pens) that remained on trial until the final two harvest dates were used for statistical analysis.

#### 2.3.2. Growth Performance

All growth performance was calculated on a shrunk live basis (BW × 0.96), and the pen was the experimental unit for growth performance, efficiency of dietary net energy utilization, and continuous carcass data. Lambs were weighed individually at processing on d −3, 1, 7, 14, 21, 56, and 63 on a platform scale (scale readability ± 0.45 kg). Initial shrunk BW was the average of d −3 and d 1 shrunk BW. Because the slaughter capacity of the SDSU Meat Laboratory was limited to 16 hd/kill date, one lamb per pen was randomly selected for harvest following the d 56 weighing and the second lamb in the pen was harvested one week later following the d 63 weigh event. Thus, final shrunk BW for each pen was the average of d 56 and d 63 shrunk BW.

#### 2.3.3. Efficiency of Dietary Net Energy Utilization

Applied energetics measures were assessed for the cumulative feeding period and shrunk live BW was used to calculate performance-based dietary net energy (NE) to determine efficiency of dietary NE utilization. Daily energy for maintenance (EM) was calculated as: EM = 0.056 Mcal × W^0.75^, where W is the median feeding period shrunk BW [8]. Performance-based dietary NE was calculated from daily energy gain (EG; kcal/d): EG = 0.337 Mcal × ADG × W^0.75^; a mature ram size of 86 kg was used to determine a coefficient of 0.337 Mcal/d on EG equation [8]. Dry matter intake (DMI) is related to energy requirements and dietary NE for maintenance (NEm) according to the following equation: DMI = EG ÷ (0.877NEm − 0.41), and can be resolved for estimation of dietary NEm by means of the quadratic formula x = (−b ± √(b^2^ − 4ac))/2a, where a = 0.41EM, b = −0.877EM + 0.41DMI + EG, and c = −0.877DMI [19]. Dietary NE for gain (NEg) was derived from NEm using the following equation: NEg = 0.877NEm − 0.41 [19]. Observed-to-expected (O:E) NEm and NEg were a ratio of performance-based dietary NE to tabular dietary NE values [20]. Expected DMI was estimated via the following equation: DMI (kg/d) = (EM ÷ NEm) + (EG ÷ NEg) [21]. The O:E DMI was a ratio of actual DMI and expected DMI based upon observed growth performance.

#### 2.3.4. Carcass Characteristics and Empty Body Measures

Due to the limitations of the SDSU Meat Laboratory, lambs were harvested on two separate dates 7 d apart (following d 56 and d 63 weigh events). Lambs were weighed on d 56 (25 October 2021), and one lamb from each pen was randomly selected for harvest the following morning. The following week on d 63 (1 November 2021), the remaining lambs were weighed off trial and were harvested the following morning.

During harvest, the viscera were removed and weighed full to obtain a weight including digesta. Digesta was washed from the gastrointestinal tract, and the viscera were weighed again to obtain an empty weight. Weight of digesta was subtracted from the previous day’s shrunk BW to obtain a weight of the empty body (EBW).

Carcasses were weighed before chilling to obtain a hot carcass weight (HCW) and were chilled and ribbed between the 12th and 13th ribs for evaluation of ribeye area (REA), backfat thickness (BF), and body wall thickness (BWT). Flank fat streaking, leg muscling score, and overall quality grade were recorded for each carcass. Calculated yield grade equations were obtained via the USDA yield grade [22] equation for lambs: YG = 0.4 + (10 × Adjusted 12th rib fat thickness, in). Percentage red meat yield (RMY) was calculated via the following equation: RMY = 80.33 − (0.35 × BWT, mm) + (0.83 × leg conformation score) [23]. Additionally, percentage boneless closely trimmed retail cuts (BCTRC) was calculated via the following equation: BCTRC = 49.936 − (0.18695 × HCW, kg) − (0.17228 × BF, mm) − (0.13898 × BWT, mm) + (0.38022 × REA, cm^2^) [24].

### 2.4. Study 2—Rumen Morphometrics and Small Intestine Histology

#### 2.4.1. Management

Study 2 was an evaluation of rumen morphometrics and small intestine histology of serially harvested lambs subjected to the previously discussed dietary treatments. This study used all 84 lambs that were randomized for the trial. At allotment, a subset of lambs (n = 4; initial shrunk BW = 39.1 kg ± 0.4 kg) closest to the median weight of all lambs were harvested at the SDSU Meat Laboratory and used as a baseline for rumen morphometrics and small intestine histology. Lambs (n = 80; n = 16 pens; n = 5 lambs/pen) were subjected to dietary treatments as previously discussed. On d 7, 14, 21, 56, and 63 weighing events, a single lamb from each pen was randomly selected for harvest at the SDSU Meat Laboratory. Selected lambs were removed from home pens and comingled with lambs of same treatment before harvest the following morning.

#### 2.4.2. Sample Collection

Following evisceration, a rumen sample from the cranial ventral sac was collected and washed with a 0.9% sodium chloride solution to remove any feed particles and were stored in urine collection cups in a 70% ethanol solution for further analysis. Small intestine samples were collected from the descending duodenum and the distal ileum, washed with a 0.9% sodium chloride solution, trimmed into ≈4 cm × 2.5 cm sections, placed into histological embedding cassettes, and fixed in 10% neutral buffered formalin. Empty body measurements were collected as described in Study 1.

#### 2.4.3. Rumen Morphometric and Small Intestine Histological Measurements

From the stored rumen samples, a 1 cm^2^ fragment of each sample was sectioned and papillae were counted to determine papillae per square centimeter (NOP). Additionally, 12 papillae were removed from each fragment and scanned (Epson^®^ Perfection^®^ V30; Seiko Epson Kabushiki Kaisha, Tokyo, Japan). Mean papillae area (MPA) was determined using an image analysis system (Image J; National Institutes of Health, Bethesda, MD, USA). Rumen wall absorptive surface area expressed in cm^2^ (ASA) was calculated as follows: 1 + (NOP × MPA) − (NOP × 0.002), where 1 represents the 1 cm^2^ fragment of rumen and 0.002 represents the estimated basal area of papillae in cm^2^ [25].

Duodenum and ileum samples were transported to the SDSU Animal Disease Research and Diagnostic Laboratory for histological staining. As described previously, the samples had been fixed in 10% neutral buffered formalin. The samples were cross-sectioned, embedded in paraffin, and stained with hematoxylin and eosin for microscopic evaluation. Histological slides were scanned and digitalized with a commercial glass slide scanner (MoticEasyScan^®^ Pro 6; Motic China Group Co., Ltd., Xiamen, China) and digital images were analyzed for villus height, crypt depth, villi-to-crypt ratio, and mucosal thickness with an image analysis system (Image J Version 1.53s; National Institute of Health).

#### 2.4.4. Health Management

Health outcomes for the studies were characterized as: musculoskeletal (lameness), gastrointestinal (bloat), respiratory (pneumonia), other (pink-eye, etc.), removals (includes animals found dead), and general (dead). Two lambs from the CON treatment died from acidosis and grain overload as diagnosed from the SDSU Animal Disease Research and Diagnostic Laboratory. One lamb from the CON treatment and three lambs from the BZ treatment were treated for signs of early acidosis or polio with peptobismuth and thiamine. One lamb from the CON treatment was treated for lethargy and apparent hindquarter pain therapeutically with 5 mL of a Vitamin B complex, 1 mL dexamethasone, and electrolytes in water; following no improvement from therapeutic treatment, lamb was removed from study and treated for suspected tail head infection. Growth performance and efficiency of dietary net energy for the pen of the removed wether was calculated with the data for the animal until the date of removal and was then calculated with the data from the remaining wether in the pen.

### 2.5. Statistical Analysis

#### 2.5.1. Study 1

Feedlot performance and continuous carcass data for Study 1 were analyzed as a randomized complete block design with the pen as the experimental unit. Data were analyzed with fixed effect of treatment and weight block. For model analysis, the MIXED procedure of SAS (SAS 9.4; SAS Institute, Cary, NC, USA) was used and least square means were generated for each treatment with the LSMEANS option of SAS. Categorical carcass data for Study 1 were analyzed with the individual wether as the experimental unit. The fitted model was analyzed with the Fisher’s exact test via the FREQ procedure of SAS to determine differences between treatments. Differences between treatments were determined at an *p* ≤ 0.05, and tendencies were denoted at 0.05 < *p* ≤ 0.10.

#### 2.5.2. Study 2

Rumen morphometrics and small intestine histological measurements in Study 2 were analyzed as a randomized complete block design as a factorial scheme with individual animal as the experimental unit with fixed effects of treatment, harvest date, their interaction, and random effect of weight block. If the interaction of treatment and harvest date was not significant, then the main effect means were evaluated. The model was analyzed with the MIXED procedure of SAS with harvest date as a repeated measure and a first-order autoregressive covariance structure. Linear and quadratic orthogonal contrasts for harvest date were conducted to determine differences of gastrointestinal health measurements over the feeding period. Differences between treatments were determined at *p* ≤ 0.05, and tendencies were denoted at 0.05 < *p* ≤ 0.10.

## 3. Results

### 3.1. Study 1—Growth Performance and Efficiency of Dietary Net Energy Utilization

Results for growth performance and efficiency of dietary net energy utilization are reported in Table 3. No differences (*p* ≥ 0.26) were observed for final BW, DMI, ADG, or feed conversion efficiency. Additionally, no differences (*p* ≥ 0.54) were observed for performance-based dietary NEm and NEg or observed-to-expected DMI, NEm, and NEg.

### 3.2. Study 1—Empty Body and Carcass Characteristics

Results for empty body and continuous carcass measures are reported in Table 4. No differences (*p* ≥ 0.16) were observed for empty body weight, hot carcass weight, 12th rib backfat thickness, ribeye area, and USDA yield grade. Body wall thickness was 10.5% greater (*p* = 0.02) for BZ-fed lambs than CON lambs. Additionally, BZ-fed lambs had a 2% decrease (*p* = 0.03) in calculated BCTRC compared to CON lambs. Furthermore, BZ-fed lambs had a 1% decrease (*p* = 0.03) in calculated red meat yield compared to CON lambs. Results for categorical carcass data are reported in Table 5. No differences (*p* ≥ 0.59) were observed between treatment groups for flank fat streaking, leg score, quality grade, or liver abscess prevalence.

### 3.3. Study 2—Rumen Morphometrics

The results for rumen morphometrics for Study 2 are reported in Table 6. No treatment × harvest day interaction was observed (*p* ≥ 0.23) for rumen papillae count, mean papillae area, rumen absorptive surface area, or papillae area as a percent of absorptive surface area. Additionally, no difference (*p* ≥ 0.31) was observed between treatment groups for all measurements. Linear effects (*p* < 0.01) for harvest date were observed for mean papillae area, absorptive surface area, and papillae area as a percent of absorptive surface area. Quadratic effects (*p* < 0.01) of harvest date were observed for rumen papillae count, absorptive surface area, and papillae area as a percent of absorptive surface area.

### 3.4. Study 2—Small Intestine Histology

The results for small intestine histology for Study 2 are reported in Table 7. No treatment × harvest day interaction was observed (*p* ≥ 0.23) for duodenal mucosal thickness, ileal villi height, ileal crypt depth, and villi height-to-crypt depth ratio. Additionally, no treatment differences (*p* ≥ 0.26) were observed for any small intestine histological measurements. Linear effects (*p* < 0.01) for harvest date were observed for duodenal mucosal thickness, ileal villi height, and villi height-to-crypt depth ratio. A quadratic effect (*p* < 0.01) for harvest date was observed for duodenal mucosal thickness.

## 4. Discussion

No measured variables for live growth performance differed between BZ-supplemented and CON wethers. It has previously been reported that during the suckling period, supplementation with sodium butyrate improved lamb growth performance; however, during the fattening period, no differences were observed [6]. During the current study, lambs were not supplemented with BZ until trial initiation, so growing period growth performance could not be evaluated. However, at the same level of supplementation (2 g BZ/kg DM), feedlot steers from a previous study in our lab group had improved growth performance in the receiving period while being transitioned to a finishing diet [18]. From these studies, it appears that supplementation with butyric acid is most effective prior to the finishing phase or during transition to finishing diets. By supplementing butyrate during the suckling period or prior to the finishing phase, it has been reported that rumen papillae density and length are increased compared to controls [6,26]. The diets of ruminants prior to weaning are primarily based on milk and forages which stimulate rumen growth and musculature but poorly stimulate rumen epithelial papillae development [27]. During this period, supplemental butyric acid helps to develop the rumen epithelium and papillary growth which would have marked improvements in growth performance compared to ruminants on concentrate diets which would already have mature papillae. Thus, benefits from supplementary butyric acid would be less evident as noticed in the current study. As days on feed progressed, rumen morphometric and small intestine histological measures changed independently of dietary treatment. During the study, rumen papillae count decreased linearly, while mean papillae area, absorptive surface area, and papillae area as a percentage of absorptive surface area increased linearly. Additionally, ileal villi height and ileal villi height/crypt depth ratio linearly increased over the duration of the trial. This is likely caused by the abrupt change from a high forage to a concentrate-based diet which has been associated with an increase in total SCFA production [28,29]. The results from the current study are not surprising because prior to study initiation, all lambs were fed a hay-based diet and transitioning to a corn-based diet would increase total SCFA concentrations, leading to rumen papillae and intestinal villi growth.

Only minimal differences were observed for empty body and carcass measures for BZ-supplemented lambs compared to controls. While not statistically significant but approaching a tendency, empty body weight of wethers supplemented with BZ was numerically increased by 3% compared to controls. Similar results were realized in a feedlot steer trial conducted in our lab group, indicating that the supplementation of BZ may increase the empty body weight of supplemented ruminants [18]. Additionally, body wall thickness was increased by 10% while calculated boneless closely trimmed retail cuts and calculated red meat yield were decreased by 2% and 1%, respectively, in BZ-supplemented wethers. Similar results were realized during a beef feedlot study conducted in our lab group, indicating that supplementation of ruminants with butyric acid may increase carcass fatness resulting in decreased carcass cutability measures [18]. Butyric acid can be utilized by nearly all tissues in the body, and in peripheral tissues, butyric acid may be used in lipogenesis [30]. Future research should investigate the use of exogenous butyric acid and compositional changes in lamb carcasses.

## 5. Conclusions

For lambs abruptly transitioned from a forage-based diet to a grain-based diet, encapsulated butyric acid and zinc had only minimal effects on carcass characteristics and no effects on rumen and small intestinal health. However, days on feed (harvest date) affected rumen morphometrics and small intestine histology. Future research should be conducted to investigate the effects of supplemental butyric acid and zinc on lambs prior to finishing diet transition to determine if carryover effects from the supplementation period may improve growth performance and gastrointestinal health during diet transition.

## Figures and Tables

**Table 1:** Pre-study diet formulation and composition for wethers subjected to dietary grain challenge and supplemented with 0 g/kg or 2 g/kg encapsulated butyric acid and zinc.

Ingredient ^1^	Pre-Study Diet
Oat Hay, %	57.5
Grower Pellet ^2^, %	27.5
DDGS, %	15.0
Dry Matter, % ^3^	87.4
Crude Protein, % ^3^	13.7
Net Energy for Maintenance, Mcal/kg^3^	1.65
Net energy for Gain, Mcal/kg^3^	0.95

^1^ All ingredients on a dry matter basis except dry matter. ^2^ Grower pellet contained (dry matter basis) 47.29% soybean hulls, 39.35% soybean meal, and 13.36% supplement premix which provided 1.91% Ca, 1.64% NaCl, 0.01% Mg, 151.83 ppm Fe, 121.47 ppm Mn, 180.68 ppm Zn, 1.01 ppm Co, 6.98 ppm I, 2.55 ppm Se, 16,167.81 IU/kg Vitamin A, 4527.14 IU/kg Vitamin D3, 813.39 IU/kg Vitamin E, and 270 mg/kg decoquinate (Zoetis, Parsippany, NJ, USA) to the grower pellet. ^3^ Tabular nutrient and energy values from (Preston, 2016).

**Table 2:** Study diet formulation and composition for wethers subjected to dietary grain challenge and supplemented with 0 g/kg or 2 g/kg encapsulated butyric acid and zinc (BZ).

Ingredient ^2^	Diet ^1^	
	CON	BZ
Whole Corn, %	65.5	65.5
Pelleted Soy Hulls, %	5.0	4.8
Grower Pellet ^3^, %	28.6	28.6
Soy Oil, %	0.9	0.9
BZ, %	0	0.2
DM ^4^, %	88.7	88.8
CP ^4^, %	14.1	14.1
NE_m_ ^4^, Mcal/kg	2.02	2.02
NE_g_ ^4^, Mcal/kg	1.33	1.33

^1^ CON diet contained 0 g/kg BZ and BZ diet contained 2 g/kg BZ. ^2^ All ingredients on a dry matter basis except dry matter. ^3^ Grower pellet contained (dry matter basis) 47.29% soybean hulls, 39.35% soybean meal, and 13.36% supplement premix which provided 1.91% Ca, 1.64% NaCl, 0.01% Mg, 151.83 ppm Fe, 121.47 ppm Mn, 180.68 ppm Zn, 1.01 ppm Co, 6.98 ppm I, 2.55 ppm Se, 16,167.81 IU/kg Vitamin A, 4527.14 IU/kg Vitamin D3, 813.39 IU/kg Vitamin E, and 270 mg/kg decoquinate (Zoetis, Parsippany, NJ, USA) to the grower pellet. ^4^ Tabular nutrient and energy values from (Preston, 2016).

**Table 3:** Study 1: Live growth performance of wether lambs subjected to dietary grain challenge and supplemented with 0 g/kg or 2 g/kg encapsulated butyric acid and zinc (BZ).

Item	Dietary Treatment ^1^		SEM	*p*-Value
	CON	BZ		
Pens, n	8	8	-	-
Wethers, n	40	40	-	-
Growth performance ^2^				
Initial body weight, kg	38.6	39.1	-	-
Final body weight, kg	52.0	53.0	0.60	0.26
Dry matter intake (DMI), kg	1.49	1.52	0.018	0.34
Average daily gain (ADG), kg	0.224	0.235	0.0104	0.49
ADG ÷ DMI (G:F)	0.150	0.155	0.0059	0.61
Observed diet net energy (NE), Mcal/kg				
Maintenance (NE_m_)	1.97	2.01	0.046	0.54
Gain (NE_g_)	1.32	1.35	0.040	0.54
Observed/Expected				
DMI	1.02	1.00	0.028	0.58
Dietary NE_m_	0.97	1.00	0.023	0.54
Dietary NE_g_	0.99	1.02	0.030	0.54

^1^ CON diet contained 0 g/kg BZ and BZ diet contained 2 g/kg BZ. ^2^ Growth performance calculated on a 4% shrunk live basis.

**Table 4:** Study 1: Empty body and continuous carcass measures of wether lambs subjected to dietary grain challenge and supplemented with 0 g/kg or 2 g/kg encapsulated butyric acid and zinc (BZ).

Item	Dietary Treatment ^1^		SEM	*p*-Value
	CON	BZ		
Pens, n	8	8	-	-
Wethers, n	15	16	-	-
Days on feed	59.5	59.5	-	-
Empty Body and Carcass Traits				
Empty body weight, kg	47.6	49.0	0.650	0.19
Hot carcass weight, kg	28.3	29.0	0.37	0.28
12th rib backfat thickness, mm	6.11	6.99	0.472	0.23
Body wall thickness, mm	20.32	22.45	0.436	0.02
Ribeye area, sq cm	15.12	14.44	0.312	0.16
USDA yield grade ^2^	2.81	3.15	0.186	0.23
Boneless closely trimmed retail cuts ^3^, %	46.52	45.73	0.209	0.03
Red meat yield ^4^, %	76.07	75.48	0.149	0.03

^1^ CON diet contained 0 g/kg BZ and BZ diet contained 2 g/kg BZ. ^2^ USDA yield grade = 0.4 + (10 × Adjusted 12th rib fat thickness, in) [22]. ^3^ Boneless closely trimmed retail cuts = 49.936 − (0.18695 × HCW, kg) − (0.17228 × BF, mm) − (0.13898 × BWT, mm) + (0.38022 × REA, cm^2^) [24]. ^4^ Red meat yield = 80.33 − (0.35 × body wall thickness, mm) + (0.83 × leg conformation score) [23].

**Table 5:** Study 1: Categorical carcass data of wether lambs subjected to dietary grain challenge and supplemented with 0 g/kg or 2 g/kg encapsulated butyric acid and zinc (BZ).

Item	Dietary Treatment ^1^		SEM	*p*-Value
	CON	BZ		
Pens, n	8	8	-	-
Wethers, n	15	16	-	-
Days on feed	59.5	59.5	-	-
Flank fat streaking				
Low choice, %	26.67	25.00	-	1.00
Average choice, %	73.33	68.75		
High choice, %	0.00	6.25		
Leg score				
Low choice, %	53.33	50.00	-	1.00
Average choice, %	46.67	50.00		
Quality grade				
Low choice, %	53.33	37.50	-	0.59
Average choice, %	46.67	56.25		
High choice, %	0.00	6.25		
Liver health				
Normal, %	60.00	68.75	-	0.72
Abscess, %	40.00	31.25		

^1^ CON diet contained 0 g/kg BZ and BZ diet contained 2 g/kg BZ.

**Table 6:** Study 2: Rumen morphometrics of serially harvested wether lambs subjected to dietary grain challenge and supplemented with 0 g/kg or 2 g/kg encapsulated butyric acid and zinc (BZ).

Item	Dietary Treatment ^1^		SEM	*p*-Value ^2^
	CON	BZ		
Pens, n	8	8	-	-
Wethers, n	37	40	-	-
Rumen papillae count ^3^, n				
d 0 ^4^	54.95	-	3.923	2 *, 4 *, 5 *
d 7	43.62	49.26		
d 14	53.24	48.34		
d 21	51.99	52.21		
d 56	45.63	46.85		
d 63	40.94	42.94		
Mean papillae area ^3^, cm^2^				
d 0 ^4^	0.180	-	0.0251	2 *, 4 *
d 7	0.156	0.165		
d 14	0.246	0.248		
d 21	0.238	0.232		
d 56	0.253	0.313		
d 63	0.337	0.292		
Absorptive surface area, cm^2^/cm^2^ of rumen wall ^3^				
d 0 ^4^	9.98	-	1.153	2 *, 4 *, 5 *
d 7	7.06	8.94		
d 14	13.58	12.85		
d 21	12.87	12.57		
d 56	11.66	15.34		
d 63	13.89	13.10		
Papillae area % of ASA ^3^				
d 0 ^4^	90.54	-	0.9544	2 *, 4 *, 5 *
d 7	85.97	87.94		
d 14	93.24	92.68		
d 21	92.66	92.83		
d 56	91.87	93.35		
d 63	92.90	92.77		

^1^ CON diets contained 0 g/kg BZ and BZ diets contained 2 g/kg BZ. ^2^ *p*-values = 1: Trt; 2: DOF; 3: Trt × DOF; 4: DOF Linear; 5: DOF Quadratic; * *p* ≤ 0.05; ^3^ Rumen morphology measures were collected and calculated [25]. ^4^ d 0 values are baseline values from the 4 hd harvest on d 0 of study.

**Table 7:** Study 2: Small intestine histology of serially harvested wether lambs subjected to dietary grain challenge and supplemented with 0 g/kg or 2 g/kg encapsulated butyric acid and zinc (BZ).

Item	Dietary Treatment ^1^		SEM	*p*-Value ^2^
	CON	BZ		
Pens, n	8	8	-	-
Wethers, n	37	40	-	-
Duodenal mucosal depth, μm				
d 0 ^3^	562.32	-	67.008	2 *, 4 *, 5 *
d 7	520.97	553.38		
d 14	693.73	789.51		
d 21	683.07	682.14		
d 56	453.44	468.55		
d 63	592.76	535.96		
Ileal villi height, μm				
d 0 ^3^	374.48	-	23.700	2 *, 4 *
d 7	385.03	421.01		
d 14	453.65	409.32		
d 21	386.95	447.73		
d 56	460.29	492.28		
d 63	467.13	467.31		
Ileal crypt depth, μm				
d 0 ^3^	245.50	-	25.415	NS
d 7	236.27	272.13		
d 14	277.17	259.29		
d 21	237.77	256.71		
d 56	263.51	251.06		
d 63	292.90	268.59		
Villi height: crypt depth				
d 0 ^3^	1.53	-	0.127	2 *, 4 *
d 7	1.61	1.50		
d 14	1.61	1.55		
d 21	1.61	1.71		
d 56	1.79	1.93		
d 63	1.58	1.69		

^1^ CON diets contained 0 g/kg BZ and BZ diets contained 2 g/kg BZ. ^2^ *p*-values = 1: Trt; 2: DOF; 3: Trt × DOF; 4: DOF Linear; 5: DOF Quadratic; * *p* ≤ 0.05; NS = not significant (*p* > 0.10). ^3^ d 0 values are baseline values from the 4 hd harvest on d 0 of study.

## Data Availability

Data available on request to Z.K.S.
